# Complete Genome Sequence of *Pantoea stewartii* RON18713 from Brazil Nut Tree Phyllosphere Reveals Genes Involved in Plant Growth Promotion

**DOI:** 10.3390/microorganisms11071729

**Published:** 2023-06-30

**Authors:** Rodrigo Theodoro Rocha, Felipe Marques de Almeida, Marília C. R. Pappas, Georgios Joannis Pappas, Karina Martins

**Affiliations:** 1 Department of Cell Biology, University of Brasília, Brasília 70910-900, DF, Brazil; theodoro.biotec@gmail.com (R.T.R.); almeidafmarques@gmail.com (F.M.d.A.); 2 EMBRAPA Genetic Resources and Biotechnology, Brasília 70770-917, DF, Brazil; marilia.pappas@embrapa.br; 3 Department of Biology, Federal University of São Carlos, Sorocaba 18052-780, SP, Brazil

**Keywords:** *Pantoea stewartii* subsp. *indologenes*, Brazil nut tree, Amazon rainforest, genome, plant–microbe interaction, type III secretion system

## Abstract

The Amazonian rainforest is a hyper-diverse ecosystem in the number of species and the myriad of intertaxon relationships that are mostly understudied. In order to characterize a dominant and economically important Amazonian species, the Brazil nut tree (*Bertholletia excelsa* Bonpl.), at the genome level, wegenerated high-coverage long-read sequencing data from the leaves of a single individual. The genome assembly revealed an unexpected discovery: two circular contigs that could be assigned to the chromosome and a plasmid of a *Pantoea stewartii* strain. Comparative genomics revealed that this strain belongs to the *indologenes* subspecies and displays high synteny with other strains isolated from diseased leaves of the neotropical palm *Bactris gasipaes* Kunth. Investigation of pathogenicity-related genes revealed the absence of the entire type III secretion system gene cluster in the plasmid, which was otherwise highly similar to a plasmid from an isolate known to cause disease in *Dracaena sanderiana* Mast. In contrast, several genes associated with plant-growth promoting traits were detected, including genes involved in indole-3-acetic acid (IAA) production, phosphate solubilization, and biosynthesis of siderophores. In summary, we report the genome of an uncultivated *P. stewartii* subsp. *indologenes* strain associated with the Brazil nut tree and potentially a plant growth-promoting bacteria.

## 1. Introduction

The Amazon rainforest is a hyper-diverse ecosystem, host to a plethora of species dynamically interacting in multi-trophic networks. Plants are its most conspicuous denizens, given their sheer number and species richness. Another fundamental component in this ecosystem, yet largely unexplored, is the rich assemblage of microbes intimately associated with plant hosts, the phytomicrobiome. From parasitism to mutualism, microbial communities dynamically adapt and interact with the host plant at species and even at genotype level, in concert with the plant holobiont concept [1].

Endophytic microorganisms (bacteria and fungi) are ubiquitous in the plant holobiont and are associated with several plant tissues, mainly the root system (rhizosphere) but also found in the leaves (phyllosphere) and the vascular system [2,3]. It is well documented that endophytes can be beneficial, inducing plant growth through several mechanisms, such as promoting the assimilation of nutrients (inorganic phosphate, iron, and nitrogen), producing phytohormones and secondary metabolites used for fending off pathogens and adapting to stress conditions [3,4,5].

Few studies have been conducted to characterize phytomicrobiomes in Amazonian tree species. A study of samples of cultivated and non-cultivated endophytic bacteria from the phyllosphere of *Paullinia cupana* Kunth, contrasting with anthracnose resistance, revealed significant community composition differences, which raised the hypothesis that some bacteria were helping the plant to mitigate the infection with *Colletotrichum* sp. [6]. Additionally, for the same tree species, vulnerability to a different fungus (*Fusarium decemcellulare*) was correlated with community disparities, especially the exclusive presence of the genus *Pantoea* in the leaves of resistant trees [7].

A study involving the cultivation of endophytic bacteria isolated from the roots of the Brazil nut tree (*Bertholletia excelsa* Bonpl.) identified several isolates that may promote plant growth through different mechanisms, including inorganic phosphate solubilization, potential nitrogen fixation, and production of indole compounds. In particular, a *Pantoea* sp. isolate produced the highest levels of indole compounds when grown in media supplemented with tryptophan. This amino acid is the biosynthetic precursor of indole-3-acetic acid (IAA) in bacteria [8]. By association, a fraction of the detected indole compounds might be in the form of IAA, a crucial phytohormone for plant growth and development. As a result, the authors hinted at utilizing some isolates to inoculate Brazil nut seeds to promote germination and seedling growth [9].

Members of the *Pantoea* species complex are associated with plants, and some strains are classified as plant growth-promoting bacteria (PGPB) and used as biofertilizers and biocontrol agents [10,11,12,13]. However, some species, particularly *Pantoea stewartii*, are well-characterized plant pathogens responsible for causing Stewart’s wilt disease in corn and other severe damages in economically important crops [14,15,16]. Infective strains contain several pathogenicity factors, especially the type III secretion system (T3SS) for injection of pathogenicity effectors into plants, and they produce a special exopolysaccharide (EPS) called Stewartan, which promotes the formation of dense biofilms [17,18]. This beneficial/pathogenic duality can be effectively assessed using intrinsic genomic features, shedding light on whether a strain is a challenger or a partner of the plant holobiont [13,19].

To characterize a dominant and emblematic species of the Amazonian flora, the Brazil nut tree (*B. excelsa*, family: Lecythidaceae), we sequenced the genome of a single tree by extracting DNA from healthy-looking and undamaged leaves. After the genome assembly, two highly contiguous sequences were of bacterial origin and later identified as a high-quality genome from a *P. stewartii* strain. We report the genome characterization of this bacterium recovered from the Brazil nut tree phyllosphere and provide circumstantial evidence of a mutualistic association with the plant host.

## 2. Materials and Methods

### 2.1. Isolation of B. excelsa Leaves and DNA Sequencing

The biological sample was an adult native *B. excelsa* individual located at 8°48.537′ S, 63°51.213′ W, in Porto Velho, Rondônia state, Brazil, and the collection occurred in June 2018, corresponding to the dry season in the region (NCBI BioSample ID: SAMN24607203). Owing to the tree’s height (≈40 m), rappelling gear was required to collect some branches from the intermediate layer of the crown.

Healthy-looking and undamaged young leaves were chosen from the branches and cleaned with moist paper towels to remove external debris, but they were not surface sterilized with additional chemical treatments [20]. Individual leaves were kept fresh until DNA extraction by placing them on a stack of moist paper towels and inserted into plastic zipper storage bags at 4 °C.

Total DNA was extracted from 1 g of fresh leaves and used as input for sequencing using the PacBio^®^ Sequel platform. Library preparation followed PacBio’s protocol, except that DNA was not sheared (50–70 kb after extraction), and the end-repair step was extended to 30 min. The resulting SMRTbell library™ was sequenced in 10 SMRT™ cells using v2.1 chemistry. Sequencing reads have been deposited in the NCBI sequence read archive (SRA) under the accession number SRR20073500.

### 2.2. Genome Assembly

Genome assembly was performed using Canu v1.8 using default parameters [21]. Contaminant screening was performed using Kraken 2 v2.1.2 [22] and a standard database (RefSeq archaea, bacteria, viral, plasmid, human, UniVec_Core, release: 13 March 2023) and with BLAST v2.13.0+ [23] searches against the NCBI non-redundant nucleotide database (downloaded: 15 May 2023). The circularity of the assembled contigs was assessed using Circlator v1.5.5 [24]. Assembly quality was assessed with the expected gene-space representation using BUSCO v4.1.0 [25] with the enterobacterales dataset (enterobacterales_odb10.2019-04-24).

Bacterial genome assemblies were deposited in the GenBank database under the accession numbers CP116285 (chromosome) and CP116286 (plasmid).

### 2.3. Comparative Genomics

The average nucleotide identity (ANI index) was calculated using the referenceseeker software v1.6.3 [26] against the pre-built National Center for Biotechnology Information (NCBI) bacteria RefSeq database (downloaded: 13 January 2023). The whole-genome phylogenetic classification was performed using the TYGS web server [27] and M1CR0B1AL1Z3R [28], using sequences obtained from the NCBI genomes database (Available from: https://www.ncbi.nlm.nih.gov/data-hub/genome; last accessed 13 March 2023). Whole-genome alignments were generated using MUMmer v4.0.0 [29], and assembly sequence differences were computed using SyRIv1.6.3 [30]. Orthologous gene clusters were identified using OrthoFinder v2.5.4 [31].

### 2.4. Gene Annotation

Gene prediction and annotation were performed using Prokka v1.14.6 [32] through the use of the bacannot pipeline v2.2 (https://github.com/fmalmeida/bacannot; accessed on 4 February 2021). Genomic islands were predicted using IslandViewer 4 [33]. Electronic PCR was performed using the EMBOSS package v6.6.0 [34]. Prediction of type III secretion system effectors was performed using the Effectidor web server v1.05 [35], and the type VI secretion system gene cluster and effectors were predicted using the SecReT6 web server v3.0 [36]. Biosynthetic gene clusters (BCGs) were predicted using antiSMASH 7.0 [37].

### 2.5. Genome and Gene Visualization

Local assembly differences were visualized using plotsr v1.0.0 [38]. Circular representations of genomes were generated using circos v0.69.8 [39]. Gene cluster comparisons were created using clinker v0.0.27 [40] and the Python package pyGenomeViz v0.3.2. UpsetPlots were generated using the Python package upsetplot v0.8.0.

## 3. Results

### 3.1. Genome Assembly and Phylogenetic Classification

The DNA extracted from the leaves of an individual Brazil nut tree was sequenced using PacBio’s long-read technology at an estimated coverage of 180× (relative to the plant’s genome size). After the genome assembly, we obtained 45 scaffolds, of which 17, with sizes ranging from 27 to 45 Mb, were identified as being of plant origin, specifically the *B. excelsa* chromosomes.

The remaining 28 scaffolds (larger than 2 kb) were tested for the presence of contaminating organisms using Kraken2 [22] and BLAST searches against the NCBI non-redundant nucleotide database. Out of the 28, we found that 26 did not match any organism other than plants, as expected. In a serendipitous event, we identified two scaffolds of approximately 4.3 Mb and 260 kb that originated from the bacterial species *P. stewartii*. After conducting an additional analysis, it was discovered that these two scaffolds were circular [24], indicating that a complete assembly of prokaryotic replicons, consisting of a bacterial chromosome (larger) and a plasmid, had been achieved. Given this unexpected finding, our aim was to categorize these elements in terms of taxonomy and functional attributes based on their sequences.

**Figure 1:**
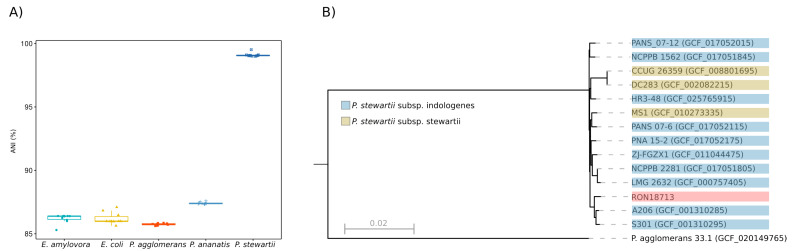
(**A**) Distribution of ANI values computed using the RON18713 chromosome as a reference against a sample of 10 genomes of selected species (x-axis) released by the NCBI; (**B**) Maximum-likelihood phylogenetic tree obtained from the M1CR0B1AL1Z3R server [28] based on the alignment of orthologous sets of genes shared by all analyzed species (3216 core genes). The red color background indicates the current studied strain.

We used the TYGS server [27] to conduct a phylogenomic analysis, in order to taxonomically classify the bacterial chromosome by searching it against the prokaryotic type strain database. To increase the number of genomes analyzed in the TYGS search, the chromosome was compared against all genomes in the NCBI bacterial RefSeq database. This was done by calculating the average nucleotide identity index (ANI) [26]. The results showed that the closest genome was from the *P. stewartii* A206 strain (GenBank assembly accession: GCF_001310285), isolated in Costa Rica from infected leaves of the neotropical palm *Bactris gasipaes* Kunth. The ANI index was 99.51, which is above the species boundary threshold (95–96%) [26]. To illustrate the ANI value distribution to delineate the species boundaries, a sample of 10 genomes from NCBI (scaffold assembly level) was drawn from selected species (accessions in Supplementary Material S1). Figure 1A shows that the ANI values for the *P. stewartii* species cluster were above 99%, while the other species had ANI values below 90%.

*P. stewartii* was subdivided into two subspecies, *stewartii* and *indologenes* [41]. A more comprehensive analysis, based on the multiple alignment of shared ortholog sets from selected *Pantoea* genomes, also posited that the studied strain has the A206 strain as its closest neighbor, along with strain S301 (GCA_001310285), which was also isolated in Costa Rica from the same plant host as A206. The resulting phylogenetic tree, derived from the concatenation of orthologous genes, revealed that these three strains are placed in a clade with several representatives of the *P. stewartii* subsp. *indologenes* subspecies (Figure 1B).

To address the paraphyly and short branch lengths in the core genes phylogenetic tree (Figure 1B), we simulated a real-time PCR assay using primers developed to distinguish between the two subspecies in question [41]. An in silico PCR analysis using the genome sequences of the selected strains in Figure 1B was performed, and the results showed that they all belonged to the *indologenes* subspecies, while the strains DC283 and CCUG 26359 were correctly assigned to the *stewartii* subspecies (Supplementary Material S2). Therefore, the results indicate that the studied strain, henceforth named *P. stewartii* RON18713, belongs to the *indologenes* subspecies.

### 3.2. Genome Assembly Metrics

The genome assembly was comparable to other *P. stewartii* representatives, ranging in size from 4.5 to 4.9 Mb and with G+C contents of 53–54% [10,42]. The bacterial chromosome was found to be composed of 4016 protein-coding sequences, 22 ribosomal RNAs, and 83 transfer RNA genes. Assembly quality assessment using BUSCO [25] indicated an overall 97.3% completeness. Despite not being the first *P. stewartii* subsp. *indologenes* genome available, our assembly was more contiguous and resolved at the chromosome level compared to the most similar genome (strain A206) (Table 1), making it a reference-quality genome for *P. stewartii* subsp. *indologenes*, similar to the current NCBI representative genome for the *indologenes* subspecies (strain ZJ-FGZX1).

Aside from the bacterial chromosome, the other identified circular contig was a ≈260 kb plasmid (GenBank: CP116286) with 230 annotated protein-coding genes (Supplementary Material S3). This replicon was highly similar, in terms of sequence identity (BLASTn 99.0% identity over 72% query cover), to one of the two reported plasmids from strain ZJ-FGZX1 (GenBank: CP049116; ≈326 kb), and the size differences are investigated later.

### 3.3. Comparative Genomic Analyses

**Figure 2:**
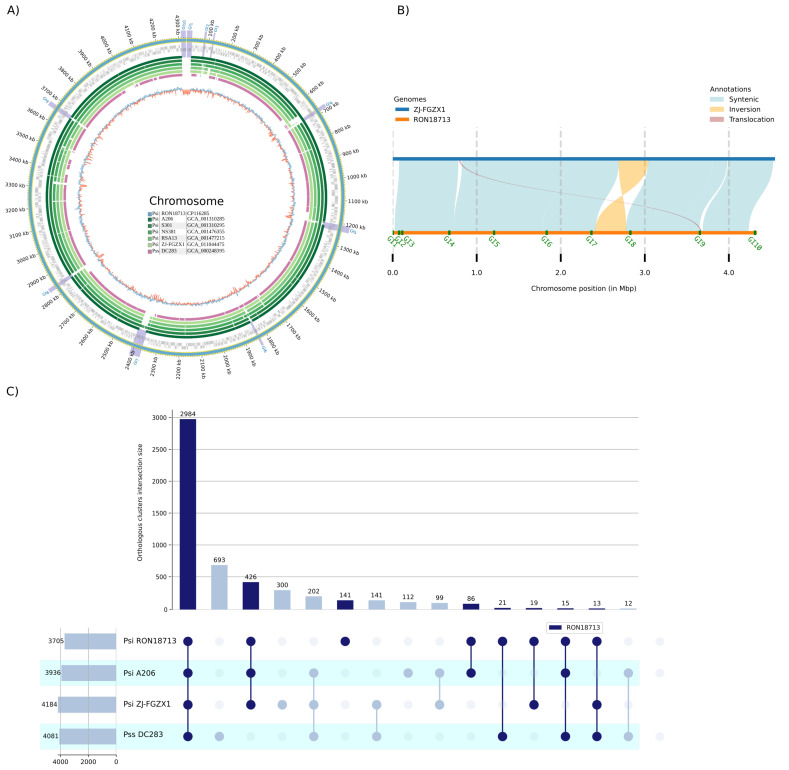
(**A**) Representation of whole-genome alignments, generated using MUMmer4, between *P. stewartii* strains isolated from plants. *P. stewartii* RON18713 chromosome (outermost ring) aligned against the closest representatives of *P. stewartii* subsp. *indologenes* and *P. stewartii* subsp. *stewartii* subspecies. Predicted genomic islands of strain RON18713 are highlighted and labeled sequentially as “GI”. (**B**) Identification of local genome sequence differences using the program SyRI [30] between *P. stewartii* RON18713 and ZJ-FGZX1. (**C**) UpsetPlot showing the number of orthologous genes shared by all analyzed species. The names of the species and the respective number of annotated proteins are shown in the leftmost horizontal bars. The number of shared orthologous groups resolved by Orthofinder2 is shown in the vertical bars. Abbreviations: Psi = *P. stewartii* subsp. *indologenes*, Pss = *P. stewartii* subsp. *stewartii*.

Whole-genome alignment using several close genomes (from Figure 1) showed high collinearity among all *P. stewartii* subsp. *indologenes* chromosomes and, to a lesser extent, to the reference genome of a *P. stewartii* subsp. *stewartii* representative, strain DC283 (Figure 2A). In addition, it is possible to observe that the alignment gap positions in *P. stewartii* RON18713 are well correlated with the predicted genomic islands for this strain. A closer inspection of the chromosomal alignment against strain ZJ-FGZX1 (Figure 2B) shows the great prevalence of syntenic blocks, except for a major inversion probably caused by the presence of two flanking genomic islands (GI7 and GI8 in Figure 2B). In fact, the presence of these genomic islands in the *P. stewartii* RON18713 chromosome seems to be associated with the genomic plasticity of *P. stewartii* strains, as they are also correlated with the majority of the structural variations observed (Figure 2B).

Furthermore, the similarity between *P. stewartii* strains extends to their entire proteomes. By using OrthoFinder2 [31], the proteomes of four strains (with a total of 16,035 proteins) were grouped into 4153 orthologous groups (OGs), with 71.9% of the OGs containing sequences from all species (Figure 2C). As expected, the strain with the greatest number of singleton OGs (693) belongs to a different subspecies (strain DC283). In addition, 141 OGs were identified as having sequences exclusive to RON18713, with 47 situated in the predicted genomic islands (Figure 2B). With regards to annotations, the great majority of RON18713 singletons (95%) had no functional category associated with them and were classified as hypothetical proteins.

### 3.4. Biosynthetic Gene Clusters

**Figure 3:**
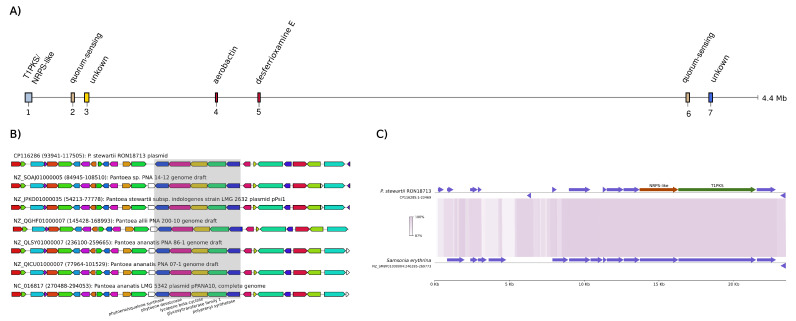
(**A**) AntiSMASH prediction of BGCs in the chromosome of RON18713. (**B**) Extended synteny of gene regions including the predicted carotenoid biosynthesis BGC (highlighted in gray) found in the plasmid of RON18713. NCBI accession numbers and genomic coordinates are on the left. (**C**) Alignment of the BGC 1 region in RON18713 and a contig from *Samsonia erythrina* (NZ_SMBY01000004). The regions are colored by the nucleotide identity, and the main biosynthetic enzymes (T1PKS and NRPS-like) are colored distinctively in BGC 1.

Based on the annotation of secondary metabolite biosynthetic gene clusters (BGC) provided by antiSMASH [37], seven potential clusters were found in the RON18713 chromosome (Figure 3A), with one located in the plasmid.

In the chromosome, the predicted BGCs (3 and 7) could not be reliably allocated to any known biosynthetic pathway, whereas the remainder could be assigned to the production of siderophores (4: aerobactin and 5: desferrioxamine E) and the quorum-sensing compound acyl-homoserine lactone (BGCs 2 and 6; Figure 3A).

Special consideration is given to chromosomal BGC 1, this ≈23.5 kb region has 98% similarity to a genome contig from the phytopathogen *Samsonia erythrina* (Figure 3C). Notably, there is no similarity between these regions and any other genome of *Pantoea* spp. It is likely that a horizontal gene transfer (HGT) occurred, as both regions are located within the predicted genomic islands. In functional terms, they are annotated as being a combination of type I polyketide synthase (T1PKS) and non-ribosomal peptide synthetase-like (NRPS-like). The T1PKS enzyme (locus_tag in Genbank entry: LZT29_0016) contains the conserved domains ketosynthase, acyltransferase, peptidyl-carrier protein, and thioesterase. The NRPS-like enzyme (LZT29_0014) encloses an inactive 4′-phosphopantetheinyl transferase (ACPS) domain, an active adenylation domain, and a phosphopantetheine acyl carrier protein domain. However, the majority of the constituent genes were annotated as hypothetical proteins and the resulting biosynthetic products are unknown.

The plasmid has only one predicted BGC (bases 93,941–117,505), which encodes a carotenoid biosynthetic operon that is conserved in various *Pantoea* species (Figure 3B). While carotenoids are not directly linked to promoting plant growth, they are thought to have significant roles in bacterial physiology, including protection against oxidative stress, mediating biofilm formation, and enabling plant colonization [3,12,43].

Other relevant gene clusters for motility, plant colonization, and biofilm formation were also identified in the chromosome of RON18713, including operons for the synthesis of flagellar components (*flgA-J*: 230,437–240,521 and *fliE-R*: 3,541,104–3,552,387) and the Stewartan EPS (*cpsAL*: 3,299,204–3,317,534), which is highly similar to the one found in the pathogenic *P. stewartii* subsp. *stewartii* strain DC283 (Accession: AF077292).

### 3.5. Virulence Factors

The Stewartan EPS and T3SS are the two primary pathogenicity factors that are recognized for contributing to the disease caused by *P. stewartii* [18]. However, Stewartan has other roles and is also present in non-pathogenic species [12].

As shown previously, the plasmid found in RON18713 is highly similar to the one found in ZJ-FGZX1 (Figure 2A). The strain ZJ-FGZX1 was shown to be the causative agent of Stewart’s wilt disease in the ornamental plant *D. sanderiana* and a T3SS complex (*Hrp-Hrc* gene cluster) present in a plasmid was directly implicated in the pathogenesis process via the injection of detrimental protein effectors into the host cells [14,15,17,44]. To investigate if the RON18713 strain contains this prototypical virulence factor, we performed a sequence similarity search using T3SS genes from ZJ-FGZX1 and other known pathogenic *P. stewartii*, but no significant hits were found in the chromosome or plasmid.

**Figure 4:**
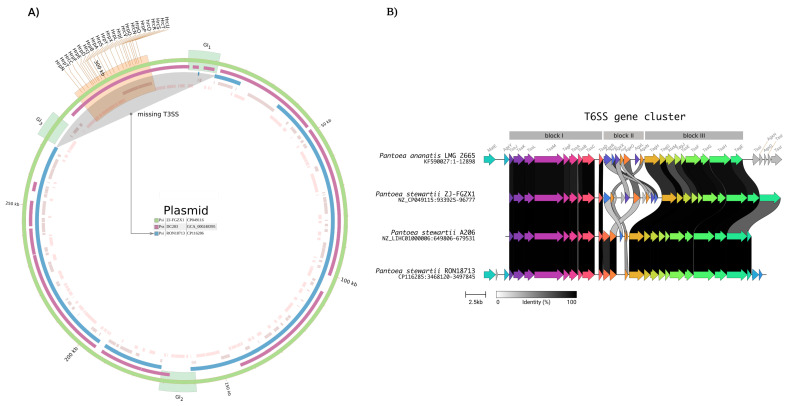
(**A**) Circos representation of the plasmid alignments using the strain ZJ-FGZX1 as reference (outermost ring). Highlighted in gray is the missing region corresponding to T3SS in RON18713 (innermost ring). (**B**) Alignment of the T6SS gene cluster from several species. Genes are represented by arrows and their links between strains are color-coded according to the identity of the resulting proteins. Blocks I to III delimit regions with high or low conservation. Abbreviations: Psi = *P. stewartii* subsp. *indologenes*, Pss = *P. stewartii* subsp. *stewartii*.

Whole-plasmid alignment revealed that the complete T3SS gene cluster is missing in RON18713, but intact in the pathogenic *P. stewartii* strains ZJ-FGZX1 and DC283 (Figure 4A). One interesting observation was that the plasmid from ZJ-FGZX1 contains two genomic islands bordering the T3SS complex and delimiting the missing region in RON18713. This deletion accounts for the size difference between the otherwise very similar plasmids. Moreover, in addition to the T3SS complex, seven predicted type III effector proteins [35] are also absent in RON18713.

Another injection apparatus complex of Gram-negative bacteria is the type VI secretion system (T6SS) that is found in both pathogenic and beneficial plant-associated bacteria [45]. Prediction of T6SS in RON18713 using SecReT6 [36] revealed the presence of a complete T6SS complex in the chromosome. A comparative analysis of the T6SS gene cluster with other strains is shown in Figure 4B. Two syntenic blocks (block I and III) display very high sequence conservation in the core genes, even for a different species, as is the case of the pathogenic *P. ananatis* LMG 2665. These conserved blocks were observed previously for several members of *Pantoea* spp. [46]. However, an abrupt break in synteny was observed in a specific region (block II) and gene conservation was only observed for the two closely related strains RON18713 and A206. Strain ZJ-FGZX1 from the same subspecies, but from distant geographical origin contains a highly divergent assortment of genes in block II, but the function of these mostly hypothetical genes remains undefined.

### 3.6. Plant Growth-Promotion Factors

While *P. stewartii* has been shown to have harmful effects on plants [14], there have been several reports indicating that certain strains of *Pantoea* spp. can have positive impacts on plant fitness and metabolism [11,19]. These impacts are attributed to the production of diverse bacterial compounds that promote plant growth through various mechanisms, such as regulating hormonal balance, acquiring nutrients, and competing against pathogens [13]. We investigated the presence of genes potentially associated with plant growth promotion using sequences from related species [12,47,48] and through manual inspection.

#### 3.6.1. Phosphate and Iron Assimilation

Table 2 lists various nutrient acquisition systems involved in phosphorus and iron uptake that are present in the chromosome. Organic gluconic acid, synthesized by extracellular glucose, is a hallmark molecule for inorganic phosphate solubilization [49]. *P. stewartii* RON18713 is apt to produce this molecule via several encoded glucose dehydrogenases (*gdh*) and the operon for the biosynthesis of the required cofactor, pyrroloquinoline quinone (PQQ). Also present in the genome are genes involved in phosphonate degradation and transport, which may further enhance the phosphate bioassimilation from the environment [47].

As for iron mobilization, there are gene clusters assigned to conserved *Pantoea* BGCs (Figure 3A) involved in the production of the hydroxamate siderophores aerobactin and desferrioxamine E (Table 2). Besides their capability to synthesize siderophores, the chromosome has genes encoding membrane transporters, which could be involved in absorbing ferrous ions or iron-bound to siderophores.

No genes involved in nitrogen fixation were found (*nif* operon), albeit being reported in some *Pantoea* strains [12].

#### 3.6.2. Phytohormones

In terms of the production of compounds directly or indirectly affecting plant hormonal balance, the presence of the indole-3-pyruvate decarboxylase gene (*ipdC*, EC:4.1.1.74) in the chromosome of RON18713 is an indication of competency to produce the phytohormone IAA, as shown in previous genomic surveys [50]. This is a key and rate-limiting enzyme in the indole-3-pyruvic acid (IPyA) pathway, which starts with the deamination of L-tryptophan to IPyA by an aromatic-amino-acid transaminase (*aatA*; EC:2.6.1.57), followed by its decarboxylation to indole-3-acetylaldehyde (IAAld) by *ipdC* [8,51]. In the last step, IAAld is oxidized to IAA catalyzed by a NAD-dependent aldehyde dehydrogenase (EC:1.2.1.3). A specific indole-3-acetaldehyde dehydrogenase (*aldA*) found in *Pseudomonas syringae* (Accession: WP_011102988) was used to search the RON18713 genome. The resulting hit (LZT29_17635) was annotated as a putative betaine-aldehyde dehydrogenase with 40.9% identity with the query sequence. Table 2 displays all the genes that are potentially associated with the IPyA pathway. In addition, RON18713 contains an annotated gene coding for an auxin efflux carrier (*aec*) that is potentially involved in the transport of the anionic form IAA out of the cell [51]. Finally, two alternative routes for IAA production, using indole-3-acetamide (IAM) and indole-3-acetronitrine (IAN) as intermediates [8,51], were not detected.

The presence of tRNA recycling genes (*miaABE*), a nucleotide 5’-monophosphate nucleosidase (LOG family), and a xanthine dehydrogenase operon (*xdhABC*), in the RON18713 strain is also highlighted in Table 2. These genes encode enzymes responsible for cytokinin biosynthesis, indicating that it may also be competent in producing this hormone class [12].

Plant-beneficial bacteria produce a diverse class of low-molecular-weight molecules, called volatile organic compounds (VOCs), which have been established as signals promoting plant growth [13,52]. Unlike the majority of growth-promoting genes, which originate from the chromosome, we identified an operon (*alsSD*) responsible for producing the VOCs acetoin and 2,3-butanediol in the plasmid of RON18713. Additionally, the chromosome encodes the biosynthetic genes for *γ*-aminobutyric acid (GABA), another relevant VOC that can be produced (Table 2).

Finally, the 1-aminocyclopropane-1-carboxylate (ACC) deaminase gene, a negative regulator of the phytohormone ethylene, found in growth-promoting *Pantoea* species [4,12] was missing in the studied strain.

## 4. Discussion

In sequencing a plant genome (*B. excelsa*), it was possible to recover two highly contiguous sequences not of plant origin and later identified as the chromosome and plasmid of a *P. stewartii* subsp. *indologenes* strain. In contrast to the traditional culture-dependent organism identification, it is not possible to confirm if the presence of this bacterium results from nonspecific sample contamination or indeed establishes a definite interaction with this plant species. In the contamination scenario, several organisms would be expected, but the sequence data did not reveal any organisms other than *P. stewartii* and *B. excelsa*. Active colonization of the foliar tissue is plausible, given the high concentration of the specific bacterial DNA source that resulted in the unambiguous genome assembly of *P. stewartii*.

*P. stewartii* species complex displays host-dependent pathogenicity outcomes [16,41]. For instance, studies have shown that Stewart’s wilt disease in maize is caused by *P. stewartii* subsp. *stewartii* but not by the *indologenes* subspecies. Conversely, reinforcing the idea of host-dependent virulence, other studies have identified the *P. stewartii* subsp. *indologenes* as the causative agent of leaf blight in rice [53], center rot in onion [54], and Stewart’s wilt disease in *D. sanderiana* [15]. One key issue is the inference of the type of biological interaction *P. stewartii* RON18713 has with *B. excelsa*: is it pathogenic or does it establish neutral/beneficial relationships with this particular host?

The strict requirement of a fully functional type II secretion system (T3SS injectisome apparatus) for virulence competence in *Pantoea* [14,17,18,44,55] was previously established. In *P. stewartii* RON18713, we detected a plasmid that is highly similar to the one found in the pathogenic strain ZJ-FGZX1. The latter encodes a complete T3SS, along with a set of neighboring T3SS effector proteins that interfere with the plant immune response and physiology. However, the *P. stewartii* RON18713 plasmid lacks the entire T3SS gene cluster and the effector genes. It is known that the acquisition of T3SS inside a pathogenicity island is likely the result of a recent event of horizontal gene transfer [16,17,56]. Noticeably, the T3SS region in the pathogenic strain *P. stewartii* subsp. *indologenes* ZJ-FGZX1 is flanked by two genomic islands, which are absent in the RON18713 plasmid. This raises the possibility that this gene cluster was gained (or accordingly lost) in a horizontal transfer event that may be the determinant of the disease onset. Given this scenario, it is unlikely that *P. stewartii* RON18713 actively promotes disease in *B. excelsa*.

Among *P. ananatis* strains, some can exhibit a pathogenic lifestyle, even when lacking T3SS genes [57]. The virulence factor encoded by the HiVir gene cluster has been reported as necessary and sufficient for *P. ananatis* pathogenicity in onion [58]. Recently, some *P. stewartii* subsp. *indologenes* strains have been associated with onion diseases, despite the absence of the HiVir gene cluster, leading to the identification of Halophos, a novel biosynthetic gene cluster critical to onion pathogenicity [59]. Notably, HiVir and Halophos clusters synthesize a phosphonate molecule catalyzed by a phosphoenolpyruvate mutase (*pepM*) core gene [59]. The lack of a homologous *pepM* gene in *P. stewartii* RON18713 genome essentially rules out the production of the phosphonate compound relevant for T3SS-independent virulence.

However, there was also evidence of a different injectisome apparatus in the genome of the tested strain. The genome contains genes that can encode a full type VI secretion system and several effectors, potentially delivering disease-causing toxins to the plant. The presence of T6SS in *Pantoea* spp. is common [46] and has been associated with pathogenicity in plants, as of *Pantoea ananatis* LMG 2665 [57], or conversely, acting against phytopathogenic bacteria [60]. T6SS in RON18713 is complete and very similar in terms of its sequences to other strains, apart from a divergent region (block II in Figure 4B) that is conserved with the most similar strain A206. It was postulated that this dissimilar region is evolutionarily plastic and a reservoir of the effector proteins that confer host specificity [46]. In the face of a missing T3SS, the pathogenic strain *P. ananatis* LMG 2665 relies upon T6SS for disease promotion in onion [57]. Nevertheless, the same apparatus displayed antibacterial effects, expanding the spectrum of T6SS [57]. Currently, the role ofT6SS is viewed primarily as establishing a competitive advantage over other bacteria, rather than as a virulence factor [45,60]. In this context, the T6SS in *P. stewartii* RON18713 could be indirectly beneficial to the plant host by antagonizing with other bacteria. This can explain the recovery of sufficient quantities of bacterial DNA to assemble its complete genome, starting from asymptomatic *B. excelsa* leaves. The high density of this specific bacterial strain would be expected of an acute infection or, alternatively, could be resultant of an effective purging system that raised the bacterial load, either at the surface or inside the leaves.

The *Pantoea* genus can be associated with plants as epiphytes or endophytes in a strictly non-pathogenic mode, with isolates of *P. ananatis*, *P. agglomerans* and *P. stewartii* being classified as plant growth-promoting bacteria and being used as biofertilizer and biocontrol agents [11]. The mechanisms of action of PGPB include the supply of nutrients to the plant (either by nitrogen fixation or phosphate solubilization), increasing iron bioavailability (through siderophore production), and the modulation of phytohormones, such as IAA and cytokinins.

Diverse systems for phosphate bioassimilation [61] are present in the *P. stewartii* RON18713 genome: (1) inorganic phosphate solubilization mediated by organic acid chelation (gluconic and 2-ketogluconic), (2) organic phosphate mineralization through degradation of phytate and phosphonate, and (3) phosphonate degradation and transport systems. The pivotal role of phosphorus in Amazon forest productivity was recently shown [62]. The association with bacteria may expand the *B. excelsa* arsenal to enhance phosphorus absorption in the generally poor Amazonian soils.

Siderophores are another class of small molecules released by microorganisms that can help host plant nutrition by scavenging the limited quantities of bioavailable iron in the environment. Moreover, the high-affinity transport systems of siderophore producers decrease iron availability, antagonizing phytopathogens [4]. Two gene clusters are responsible for the production of the hydroxamate siderophores, aerobactin and desferrioxamine E, were found in the *P. stewartii* RON18713 chromosome along with specific iron/siderophore transporters.

As with many other PGPB, the strain is competent in producing the phytohormone IAA, involved in several aspects of plant growth and development, through the indole-3-pyruvate decarboxylase (*idpC*) pathway. The absence of the alternative indole-3-acetamide (IAM) route for IAA production is noteworthy, which was only reported for pathogenic *Pantoea* strains [17]. Cytokinins (CK) are produced by pathogenic and plant-growth promoting *Pantoea* spp. strains [12]. CKs regulate a broad scope of growth and developmental processes in plants, with notable context-dependent outcomes in plant immunity, either involved in pathogenesis or inducing resistance [63]. It was shown that CK production by *P. agglomerans* strains enhanced the severity of plant gall-forming diseases, through a mechanism that increases the expression of the type III secretion system by IAA (produced by the IAM route) and CK [64]. The lack of T3SS and genes involved in its biosynthesis suggests that the bacterial CK acts as a PGPB feature, by enhancing plant immunity responses to potential pathogenic microbes [63].

Genes involved in the production of the volatile organic compounds acetoin and 2,3-butanediol were also detected in the strain. These molecules are involved in several aspects of increased plant fitness, interfering with hormonal responses, iron/sulfur uptake, and plant tolerance to biotic and abiotic stresses [52].

Finally, the biosynthetic gene clusters found in RON18713 are comparable to the most similar species genome (strain A206) and to the strain ZJ-FGZX1. However, half of the predicted BGCs could not be associated with the production of known compounds, according to antiSMASH classification. Despite the overall similarity with other strains, we identified a unique BGC in RON18713 and absent in all other known genomes of the *Pantoea* genus. It is possible that this BGC was acquired through horizontal gene transfer since it is located within a genomic island and shares over 98% identity with a region from the genome of the phytopathogen *Samsonia erythrina*. This BGC encodes a hybrid type I polyketide synthase and non-ribosomal peptide synthetase (T1PKS-NRPS) and may be involved in the production of a yet uncharacterized secondary metabolite. Ultimately, this locus may be a distinctive feature of RON18713, both to provide discriminative genetic markers and for its potential effects on the host plant or the microbiome.

This study was prompted by an unexpected discovery, and its findings are based on comparative computational analysis. Although dozens of Pantoea genomes provide phylogenomic support for various traits, the mere presence of a gene or group of genes cannot definitively prove a phenotype. It is crucial to experimentally validate the data presented here, since many unresolved issues remain, including isolation and cultivation, subspecies classification, host specificity, geographical distribution, as well a biochemical and physiological characterization of *P. stewartii* RON18713. The data provided in this study are expected to provide a foundation for future investigations into this previously unstudied strain.

## 5. Conclusions

In this report, we took advantage of sequencing data usually discarded as contamination during a eukaryotic genome sequencing project, to dissect the genome of a bacterium that was neither isolated nor cultivated by formal microbiological methods. We presented a metagenome-assembled genome (MAG) of a *Pantoea stewartii* strain obtained from the Brazil nut tree phyllosphere. The genome is of high quality and completeness, in comparison with the limited number of reference genomes currently available for *P. stewartii* subsp. *indologenes*.

Despite being unable to answer relevant questions about the biological nature of the interaction between this microorganism and *B. excelsa*, the in silico genome exploration resulted in the phylogenetic placement of the strain and provided pointers about its lifestyle. Several genes were associated with plant growth-promotion traits and the noticeable lack of virulence loci characteristic of pathogenic strains of this diverse and adaptable genus.

This is the first genomic description of a *P. stewartii* subsp. *indologenes* closely associated with *B. excelsa*, which may have adapted to a non-pathogenic mutualistic lifestyle with the plant host and that opens new avenues for exploring the uncharted diversity and varied roles of the Amazonian phytomicrobiome.

## Figures and Tables

**Table 1:** Genome assembly statistics of *Pantoea stewartii* RON18713 compared to other *P. stewartii* strains with genomes available at NCBI. The assembly lengths include all replicons for each entry. Average nucleotide identity (ANI) is measured relative to the chromosome of strain RON18713.

Strain	RON18713	ZJ-FGZX1	LMG 2632	LMG 2715
Qualifier	current study	Reference genome	Type strain	Type strain
Accession number	GCA_030064655	GCA_011044475	GCA_000757405	GCA_008801695
Subspecies	*indologenes*	*indologenes*	*indologenes*	*stewartii*
Number of contigs	2	3	35	352
Total length (bp)	4,596,865	4,982,863	4,681,235	4,916,637
Contig N50 (bp)	4,334,600	4,550,072	304,929	47,061
Plasmids	1	2	1	not resolved
ANI	-	99.09	99.08	99.06

**Table 2:** Genes associated with plant growth-promotion traits in *Pantoea stewartii* RON18713. The genome coordinates refer to the chromosome, and when there is more than one gene in the cluster, the range encompasses the entire extension.

Gene	Annotation	Gene(s) Location, Strand
**Mineral and organic phosphate solubilization**		
*gad*	gluconate 2-dehydrogenase, membrane-bound	1433796-1437636, −
*gcd*	glucose/quinate/shikimate PQQ-dependent dehydrogenase	1420776-1423166, +
*pqq*	Coenzyme PQQ synthesis cluster	4055459-4058544, −
*phoU*	Phosphate-specific transport system accessory protein	1750440-1751177, −
*pstBACS*	Phosphate ABC transporter complex	1751195-1754992, −
*agp*	3-phytase (EC:3.1.3.8)	292050-293642, −
*phnAGMP*	Phosphonate C-P lyase system components	2723515-2723895, +
*phnDE*	Phosphonate ABC transporter subunits	3447629-3449532, +
**Siderophores biosynthesis and iron transporters**		
*iucABCD*	Aerobactin gene cluster	1380722-1386465, −
*dfoJACS*	Desferrioxamine E gene cluster	1129538-1136058, −
*fhuABCD*	Fe^3+^-hydroxamate ABC transporter	964875-970778, −
*fepDC*	ABC-type Fe^3+^-siderophore transport system	3242782-3244646, +
*efeUOB*	Ferrous iron uptake system	3593902-3597190, +
**Phytohormones**		
**IAA production**		
*aatA*	Aromatic-amino-acid transaminase	1472613-1473806, −
*ipdC*	Indole-3-pyruvate decarboxylase	3032974-3034626, +
*aldA*	Aldehyde dehydrogenase	3687880-3689352, +
*aec*	Auxin efflux carrier family protein	3267773-3268732, −
**Cytokinins**		
*miaA*	tRNA dimethylallyltransferase	2227421-2228326, +
*miaB*	tRNA2-methylthio-N-6-isopentenyl adenosine synthase	584029-585414, +
*miaE*	tRNA isopentenyl-2-thiomethyl-A-37 hydroxylase	1246775-1247536, −
*xdhABC*	Xanthine dehydrogenase	3907249-3911807, +
*ppnN*	nucleotide 5’-monophosphate nucleosidase	2665271-2666635, −
**Volatile organic compounds (VOCs)**		
**Acetoin and 2,3-butanediol**		
*alsR*	Transcriptional regulator of alpha-acetolactate operon alsR	Plasmid: 18242-19123, +
*alsD*	Acetolactate decarboxylase	Plasmid: 17355-18137, −
*alsS*	Acetolactate synthase	Plasmid: 15660-17339, −
*bdh*	(S)-acetoin forming diacetyl reductase	Plasmid: 14861-15637, −
***γ*-aminobutyric acid**		
*gabD*	NAD-dependent succinate-semialdehyde dehydrogenase	3417320-3418774, +
*gabT*	4-aminobutyrate–2-oxoglutarate transaminase	3432281-3433591, +

## Data Availability

Genome assembly sequences of *P. stewartii* RON18713 have been deposited in NCBI genome database under the accession number GCF_030064655 and corresponding individual replicons in GenBank under the accession numbers CP116285 (chromosome) and CP116286 (plasmid). Sequencing reads have been deposited in the NCBI sequence read archive (SRA) under the accession number SRR20073500.

## Supplementary Materials

The following supporting information can be downloaded at https://www.mdpi.com/article/10.3390/microorganisms11071729/s1.
