# Supplementary material for: Complete Genome Sequence of Pantoea stewartii RON18713 from Brazil Nut Tree Phyllosphere Reveals Genes Involved in Plant Growth Promotion
Source: Microorganisms. 2023 Jun 30;11(7):1729. doi: 10.3390/microorganisms11071729 (PMC10383142; doi:10.3390/microorganisms11071729)
Supplement: Supplementary file 1 [file microorganisms-11-01729-s001.zip › microorganisms-2347200-supplementary.pdf]

# Supplementary material

## S1. Average nucleotide identity (ANI) comparisons

The NCBI accession numbers of the genome assemblies used to calculate the ANI score against RON18713 chromosome in Figure 1A of the paper.

| NCBI Accession | Organism Name |
|---|---|
| GCA_000005845.2 | Escherichia coli str. K-12 substr. MG1655 |
| GCA_000008865.2 | Escherichia coli O157:H7 str. Sakai |
| GCA_003697165.2 | Escherichia coli ATCC 11775 |
| GCA_003018455.1 | Escherichia coli |
| GCA_024300685.1 | Escherichia coli |
| GCA_027925825.1 | Escherichia coli |
| GCA_003018035.1 | Escherichia coli |
| GCA_027925745.1 | Escherichia coli |
| GCA_027925805.1 | Escherichia coli |
| GCA_027925765.1 | Escherichia coli |
| GCA_000091565.1 | Erwinia amylovora CFBP1430 |
| GCA_017161565.1 | Erwinia amylovora |
| GCA_015650045.1 | Erwinia amylovora |
| GCA_002803865.1 | Erwinia amylovora |
| GCA_027557715.1 | Erwinia amylovora |
| GCA_012980785.1 | Erwinia amylovora |
| GCA_012980845.1 | Erwinia amylovora |
| GCA_017161545.1 | Erwinia amylovora |
| GCA_012980825.1 | Erwinia amylovora |
| GCA_023612715.1 | Erwinia amylovora |
| GCA_019048385.1 | Pantoea agglomerans |
| GCA_943184895.1 | Pantoea agglomerans |
| GCA_021397715.1 | Pantoea agglomerans |
| GCA_001709315.1 | Pantoea agglomerans |
| GCA_017474165.1 | Pantoea agglomerans |
| GCA_021559955.1 | Pantoea agglomerans |
| GCA_029016125.1 | Pantoea agglomerans |

| NCBI Accession | Organism Name |
|---|---|
| GCA_003704305.1 | Pantoea agglomerans |
| GCA_022966105.1 | Pantoea agglomerans |
| GCA_020783495.1 | Pantoea agglomerans |
| GCA_000233595.1 | Pantoea ananatis PA13 |
| GCA_004028255.1 | Pantoea ananatis |
| GCA_025765855.1 | Pantoea ananatis |
| GCA_013364135.1 | Pantoea ananatis |
| GCA_023845475.1 | Pantoea ananatis |
| GCA_002224585.2 | Pantoea ananatis |
| GCA_026075575.1 | Pantoea ananatis |
| GCA_023611845.1 | Pantoea ananatis |
| GCA_002952035.2 | Pantoea ananatis |
| GCA_001543055.1 | Pantoea ananatis |
| GCA_011044475.1 | Pantoea stewartii |
| GCA_025765915.1 | Pantoea stewartii |
| GCA_002082215.1 | Pantoea stewartii subsp. stewartii DC283 |
| GCA_000757405.2 | Pantoea stewartii subsp. indologenes |
| GCA_008801695.1 | Pantoea stewartii subsp. stewartii |
| GCA_029433915.1 | Pantoea stewartii |
| GCA_001310285.1 | Pantoea stewartii |
| GCA_013277595.1 | Pantoea stewartii |
| GCA_025599245.1 | Pantoea stewartii |
| GCA_014218605.1 | Pantoea stewartii |

# S2. Electronic PCR for *P. stewartii* subspecies differentiation

To distinguish between *P. stewartii* subspecies, Pal et al. (2019) designed primer pairs targeting the intergenic region of the *cpsA* and *cpsB* genes, that are contained in the Stewartan biosynthesis operon (*wce-I*). The primer pair cpsF (forward) and cpsR (reverse) can amplify this region in *P. stewartii* regardless of its subspecies. However, a 182 bp deletion was found in the *indologenes* subspecies, resulting in an expected amplicon size of 193 bp. In contrast, for the *stewartii* subspecies, the expected amplicon size is 375 bp.

The authors designed another forward primer (cpsAB2313F) annealing at the divergent region to further differentiate the subspecies. When used together with cpsR, this primer is not expected to generate a PCR product for the *indologenes* subspecies. In contrast, a PCR product of 193 bp is expected for the *stewartii* subspecies. The primer sequences are:

```
cpsF          AAGGTGCCAGCCTCTCTCTG
cpsAB2313F    AGAAAACGCTGATGCCAGAC
cpsR          ACTATCCTGACTCAGGCACT
```

Pal, N., Block, C. C., & Gardner, C. A. C. (2019). A Real-Time PCR Differentiating *Pantoea stewartii* subsp. *stewartii* From *P. stewartii* subsp. *indologenes* in Corn Seed. Plant disease, 103(7), 1474–1486. https://doi.org/10.1094/PDIS-06-18-0936-RE

To distinguish between the subspecies in various *P. stewartii* strains, we conducted an electronic PCR (ePCR) analysis using the cpsF-cpsR and cpsAB2313F-cpsR primer pair combinations. We used NCBI genomes as templates and included type strains for both subspecies (LMG 2632=*indologenes* and CCUG 26359=*stewartii*). The table below displays the ePCR results obtained from the `primersearch` program of the EMBOSS package. The expected amplicon sizes described above were used to diagnose the subspecies.

| | | | Amplicon | | |
|---|---|---|---|---|---|
| Strain | Accession | Subspecies | cpsF (bp) | cpsAB2313F (bp) | Diagnostics |
| RON18719 | CP116285 | unassigned | 193 | 0 | indologenes |
| A206 | NZ_LIHC01000006 | unassigned | 193 | 0 | indologenes |
| ZJ-FGZX1 | NZ_CP049115 | indologenes | 193 | 0 | indologenes |
| LMG 2632 | NZ_JPKO01000031 | indologenes | 193 | 0 | indologenes |
| DC283 | NZ_CP017581 | stewartii | 375 | 256 | stewartii |
| CCUG 26359 | NZ_VZPF01000032 | stewartii | 375 | 256 | stewartii |

The `primersearch` results are shown below.

- *Pantoea stewartii* strain RON18719

```
  Primer name cpsF-cpsR
  Amplimer 1
      Sequence: CP116285
      Pantoea stewartii strain RON18719, chromosome
      AAGGTGCCAGCCTCTCTCTG hits forward strand at 3300577 with 0
mismatches
      ACTATCCTGACTCAGGCACT hits reverse strand at [1033725] with
0 mismatches
      Amplimer length: 193 bp

  Primer name cpsAB2313F-cpsR
      Amplimer length: 0 bp
```

- *Pantoea stewartii* strain A206

```
  Primer name cpsF-cpsR
  Amplimer 1
      Sequence: NZ_LIHC01000006 NZ_LIHC01000006
      Pantoea stewartii strain A206 scaffold2.1, whole genome
shotgun sequence.
      AAGGTGCCAGCCTCTCTCTG hits forward strand at 486043 with 0
mismatches
      ACTATCCTGACTCAGGCACT hits reverse strand at [256853] with 0
mismatches
      Amplimer length: 193 bp

  Primer name cpsAB2313F-cpsR
      Amplimer length: 0 bp
```

- *Pantoea stewartii* strain ZJ-FGZX1

```
  Primer name cpsF-cpsR
 Amplimer 1
 Sequence: NZ_CP049115 NZ_CP049115
 Pantoea stewartii strain ZJ-FGZX1 chromosome, complete genome.
 ACTATCCTGACTCAGGCACT hits forward strand at 1196988 with 0
mismatches
 AAGGTGCCAGCCTCTCTCTG hits reverse strand at [3352893] with 0
mismatches
 Amplimer length: 193 bp

 Primer name cpsAB2313F-cpsR
 Amplimer length: 0 bp
```

- *Pantoea stewartii* subsp. *indologenes* strain LMG 2632

```
    Primer name cpsF-cpsR
    Amplimer 1
        Sequence: NZ_JPKO01000031.1
        Pantoea stewartii subsp. indologenes strain LMG 2632
  Contig31, whole genome shotgun sequence
        ACTATCCTGACTCAGGCACT hits forward strand at 139980 with 0
  mismatches
        AAGGTGCCAGCCTCTCTCTG hits reverse strand at [454151] with 0
  mismatches
        Amplimer length: 193 bp

    Primer name cpsAB2313F-cpsR
        Amplimer length: 0 bp
```

- *Pantoea stewartii* subsp. *stewartii* DC283

```
    Primer name cpsF-cpsR
    Amplimer 1
        Sequence: NZ_CP017581 NZ_CP017581
        Pantoea stewartii subsp. stewartii DC283 chromosome,
  complete genome.
        ACTATCCTGACTCAGGCACT hits forward strand at 3252402 with 0
  mismatches
        AAGGTGCCAGCCTCTCTCTG hits reverse strand at [1275440] with
  0 mismatches
        Amplimer length: 375 bp

    Primer name cpsAB2313F-cpsR
    Amplimer 1
        Sequence: NZ_CP017581 NZ_CP017581
        Pantoea stewartii subsp. stewartii DC283 chromosome,
  complete genome.
        ACTATCCTGACTCAGGCACT hits forward strand at 3252402 with 0
  mismatches
        AGAAAACGCTGATGCCAGAC hits reverse strand at [1275559] with
  0 mismatches
        Amplimer length: 256 bp
```

- *Pantoea stewartii* subsp. *stewartii* strain CCUG 26359

```
    Primer name cpsF-cpsR
    Amplimer 1
        Sequence: NZ_VZPF01000032.1
        Pantoea stewartii subsp. stewartii strain CCUG 26359
  contig_0000032, whole genome shotgun sequence
        AAGGTGCCAGCCTCTCTCTG hits forward strand at 32986 with 0
  mismatches
        ACTATCCTGACTCAGGCACT hits reverse strand at [18045] with 0
  mismatches
        Amplimer length: 375 bp
```

```
    Primer name cpsAB2313F-cpsR
    Amplimer 1
        Sequence: NZ_VZPF01000032.1
        Pantoea stewartii subsp. stewartii strain CCUG 26359
  contig_0000032, whole genome shotgun sequence
        AGAAAACGCTGATGCCAGAC hits forward strand at 33105 with 0
  mismatches
        ACTATCCTGACTCAGGCACT hits reverse strand at [18045] with 0
  mismatches
        Amplimer length: 256 bp
```

# S3. RON18713 plasmid map

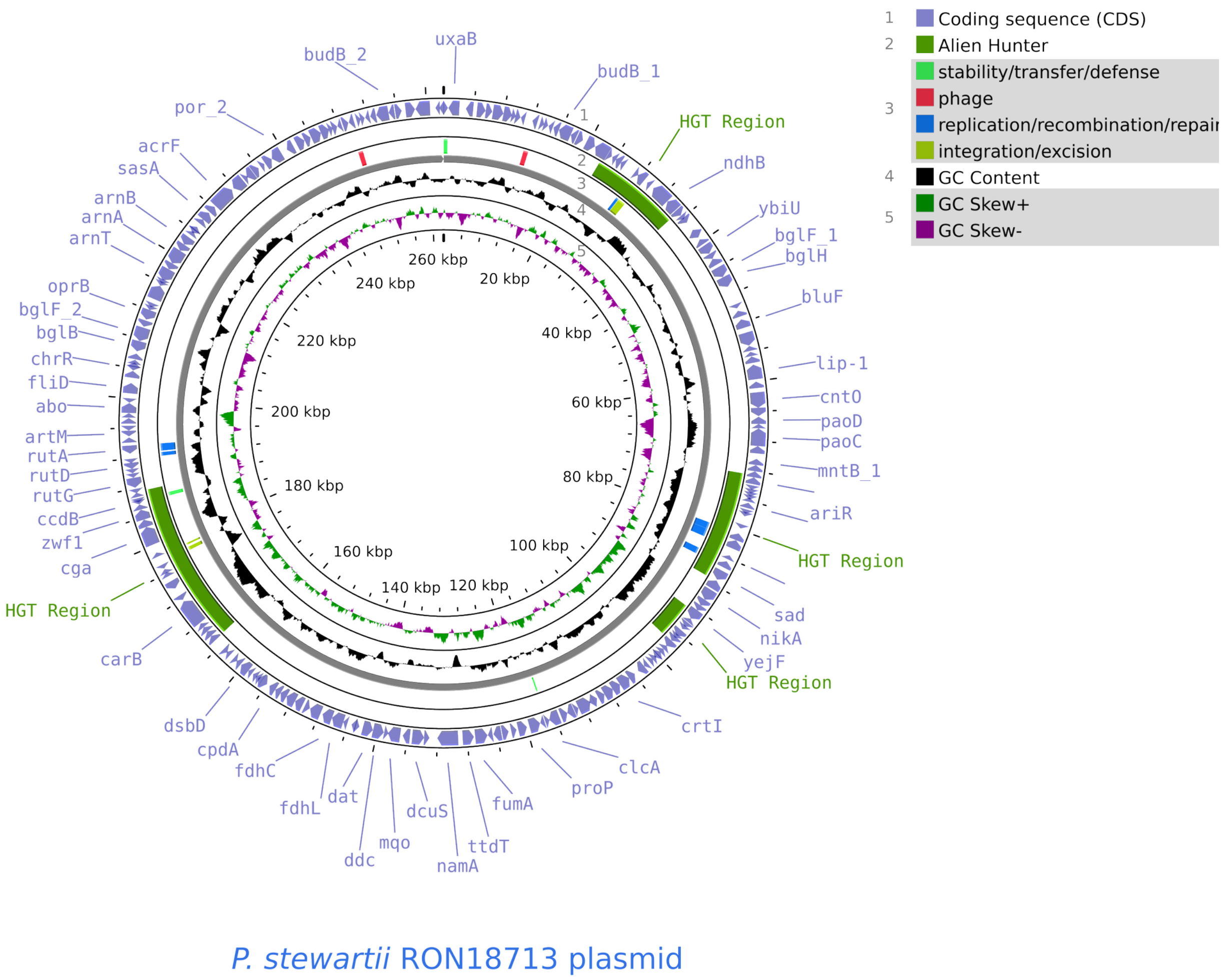


*Figure S1 - Circular representation of P. stewartii RON18713 assembled plasmid (GenBank: CP116286) generated by Proksee server (https://proksee.ca). Rings indicate the annotated coding sequences (CDS), horizontal gene transfer (HGT) events using Alien Hunter, mobile genetic elements (MGEs) using mobileOG-db (beatrix-1.6), GC content and GC skew. All annotations, except for the CDS were performed by Proksee's embedded analytical tools.*
